# Significant blunt bowel and mesenteric injury – Comparison of two CT scoring systems in a trauma registry cohort

**DOI:** 10.1016/j.ejro.2021.100380

**Published:** 2021-09-30

**Authors:** Nathalie Keller, Tobias Zingg, Fabio Agri, Alban Denys, Jean-Francois Knebel, Sabine Schmidt

**Affiliations:** a Department of Diagnostic and Interventional Radiology, Lausanne University Hospital and University of Lausanne, Lausanne, Switzerland; b Department of Visceral Surgery, Lausanne University Hospital and University of Lausanne, Lausanne, Switzerland

**Keywords:** ATMV, Abrupt termination of mesenteric vessels, AMB, Active mesenteric bleeding, AAWI, Anterior abdominal wall injury, BIPS, Bowel Injury Prediction Score, BWD, Bowel wall discontinuity, BWT, Bowel wall thickening, DBWE, Decreased bowel wall enhancement, FF, (Non-haematic) free fluid, FPP, Free pneumoperitoneum, IBMV, Irregular beading of mesenteric vessels, HP, Haemoperitoneum, MFS, Mesenteric (pericolic) fat stranding, MPP, Mesenteric pneumoperitoneum, CT, Multidetector computed tomography, sBBMI, Significant blunt bowel and mesenteric injury, SB, Small bowel, WBC, White blood cell, Polytrauma, Multidetector computed tomography, Mesentery, Intestine, small, Intestine, large

## Abstract

**Purpose:**

This study evaluated and compared two imaging-based scoring systems for the detection of significant blunt bowel and mesenteric injury (sBBMI) by emergency computed tomography (CT)

**Methods and Materials:**

We included all consecutive adult polytrauma patients admitted to our emergency department following a road traffic accident from January 2008 to June 2015, provided that intravenously contrast-enhanced whole-body CT examination was performed immediately after hospital admission. Two radiologists, blinded to patients’ outcome, reviewed the CT examinations for distinctive direct intestinal or mesenteric vascular injury and indirect signs of abdominal injury. These findings were correlated with the patients’ surgical or interventional radiology findings, autopsy, or clinical follow-up (>24 h). Two previously validated imaging-based bowel-injury scoring systems, the CT-based Faget score and the clinically and radiologically based Mc Nutt score (BIPS), were compared by applying each to our trauma cohort. Student t-test, chi-squared, and logistic regression were used in analyses.

**Results:**

Twenty-one of 752 analysed patients (2.8 %) had confirmed sBBMI. Active mesenteric bleeding, mesenteric and free pneumoperitoneum, small haemoperitoneum, non-focal bowel wall thickening, mesenteric/pericolic fat stranding, and anterior abdominal wall injury were significantly correlated with sBBMI, as did the two evaluated scoring systems (p < 0.001). However, multivariate logistic regression revealed the superiority of the Faget score to the McNutt score.

**Conclusion:**

The prevalence of sBBMI among polytrauma patients is low. Early diagnosis is necessary to avoid increased mortality. Certain CT features are pathognomic of sBBMI and must not be overlooked. Scoring systems are helpful, especially when they are based on radiological signs.

## Highlights

• Significant blunt bowel or mesenteric injury is rare in polytrauma patients.
• Certain CT features are pathognomic, but they seldom occur and may be subtle.
• Scoring systems are helpful, especially when they are based on radiological signs.

## 1 Introduction

Significant blunt bowel and mesenteric injury (sBBMI) is uncommon, occurring in 1–5 % of patients with blunt abdominal trauma [[1], [2], [3]]. Shearing forces, crush, and burst injury are the main causative mechanisms. Multidetector computed tomography (CT) is widely considered as the examination of choice for haemodynamically stable polytrauma patients [2,4,5]. Thus, the identification of early CT signs in sBBMI is essential, as patients with significant lesions need timely surgery or angioembolisation and delayed diagnosis (>8 h) may increase morbidity and mortality [[6], [7], [8], [9]]. However, these CT signs can be subtle. Moreover, it may be difficult to identify mesenteric lesions in cases of concomitant hemoperitoneum caused by solid organ laceration. Haemoperitoneum may obscure mesenteric fat oedema and/or direct signs of bowel injury. Furthermore, due to the rare incidence of sBBMI, radiologists may not be familiar with the CT findings. Thus, their early identification remains even more challenging and missed injuries remain common even in the current era of multidetector CT use [10].

Recently, two scoring systems were developed with the aim of identifying patients with sBBMI early. These scoring systems are either based on CT signs only, as published by Faget et al. [11], or combine clinical and radiological findings, as in the Bowel Injury Prediction Score (BIPS) proposed by McNutt et al. [12]. Each of these scores has been validated separately on a retrospective basis, but they have not yet been compared to each other by applying them in a large study population encountered in the clinical practice. Moreover, we do not know, if such scoring systems are really required for diagnosing sBBMI, as some of the direct CT signs are pathognomic of sBBMI.

Therefore, our aim was to retrospectively apply these two scoring systems on our own trauma register cohort and to compare their performance directly. Furthermore, we investigated whether these calculated scores are superior to the detection of isolated CT signs for immediate patient management.

## 2 Material and methods

### 2.1 Patients

This was a single-centre registry-based retrospective cohort study, prepared to conform to the Strengthening the Reporting of Observational Studies in Epidemiology (STROBE) guidelines [13]. The study was based on the prospective trauma registry of the Lausanne University hospital, including all consecutive adult patients admitted to the trauma resuscitation area of the emergency department following a road traffic accident from January 2008 to June 2015 (n = 838). Our trauma protocol follows the Advanced Trauma Life Support (ATLS) guidelines [14], adapted to the local infrastructure and resources. Shortly after arrival, each polytrauma patient undergoes a clinical examination by the attending trauma surgeon in charge. Whole-body CT with intravenously injected iodinated contrast medium is then performed in all hemodynamically stable patients.

The study protocol was approved by the institutional ethics committee (Protocol No. 2016-00928) and the requirement for informed consent was waived.

**Fig. 1:**
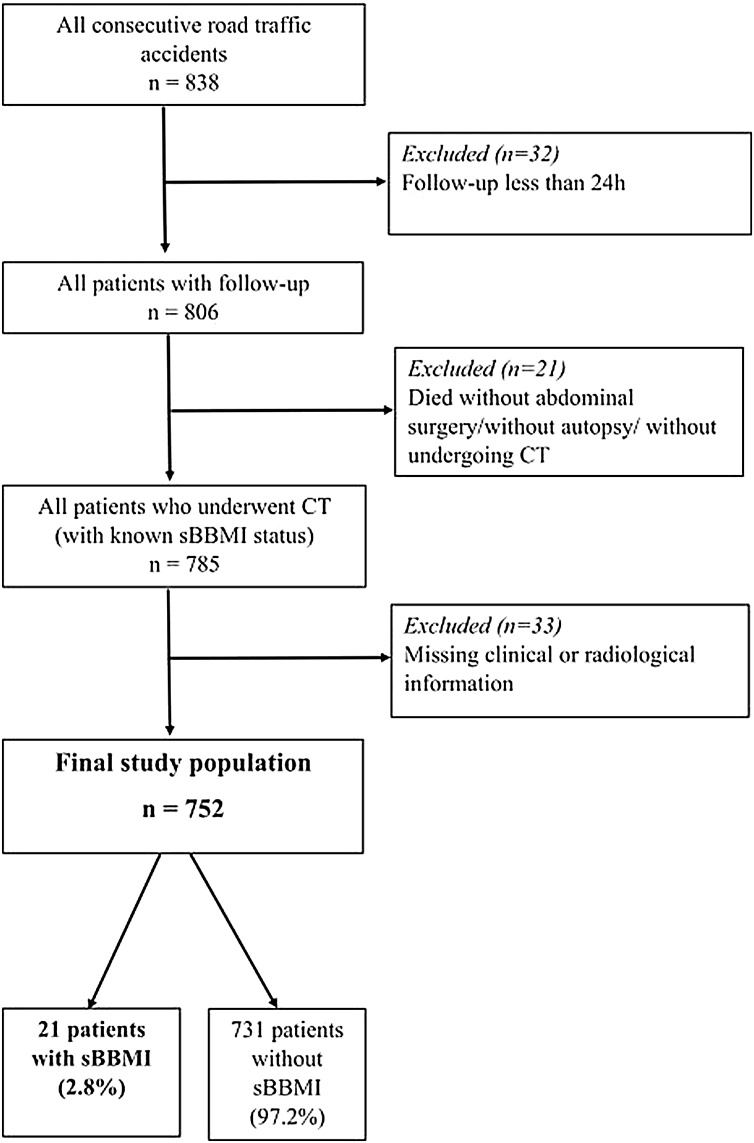
Flow chart of patient inclusion. sBBMI – significant blunt bowel and mesenteric injury

Patient inclusion is visualised in Fig.1. Our final study population consisted of 752 patients.

### 2.2 CT parameters and image analysis

Our polytrauma protocol was performed with a 64-detector row CT machine (Lightspeed VCT; 64 Pro, GE Healthcare; Milwaukee, Wisconsin, USA). In all patients, the whole abdomen was included in the data acquisition (120 kV, 300−400 mA, table speed 55 mm per rotation [0.8 s], pitch 1.375). Iodinated contrast medium (Accupaque®, Iohexol, 300 mgI/mL; GE Healthcare) was injected intravenously (volume in millilitres = body weight +30 mL) at a flow rate of 4 mL/s followed by data acquisition during venous phase (80 s), reconstructed in 1.25/1 mm and/or 2.5/2 mm axial slices. We used automatic tube current modulation in all three axes and the iterative reconstruction algorithm ASIR. Neither oral nor rectal contrast medium administration was part of our polytrauma protocol.

Two radiologists (S.Sch. and N.K:) with 20 and 5 years of expertise in abdominal imaging, respectively, jointly reviewed all CT examinations on a picture archiving and communication system (PACS) workstation (Carestream Vue, version 12.1.5; Carestream Health, Rochester NY, USA). The readers were blinded to all clinical, radiological, and surgical findings. Images were reviewed for distinctive features suggesting sBBMI as defined by the literature [2,3,11,[15], [16], [17], [18], [19], [20], [21]]. The CT features were classified into three subgroups, as shown, and in Table 1.

**Table 1:** Definition and classification of the evaluated CT signs.

CT sign	Definition
*Intestinal signs*	
Bowel wall discontinuity	Cut-off bowel wall continuity due to wall transection [2,15]
Focal bowel wall thickening (≤10 cm length) or non-focal bowel wall thickening (>10 cm length)	Small bowel or colonic wall thickening > 3 mm and > 5 mm, respectively, provided that the lumen is sufficiently distended [16,17,18]; Not considered in the case of diffuse bowel wall thickening suggesting underlying “shock bowel” [19]
Decreased bowel wall enhancement	Focal lack of the subtle physiological contrast enhancement of the bowel mucosa compared to the wall of nearby, healthy bowel loops, possibly indicating post-traumatic ischaemia [2,3]
*Mesenteric vessel signs*	
Active mesenteric bleeding	Contrast medium extravasation of mesenteric vessels during venous phase [17,18]
Irregular beading of mesenteric vessels	Abnormal vascular regularity
Abrupt vessel termination	Lack of vessel continuity [16,20]
*Extraintestinal signs*	
Mesenteric pneumoperitoneum	Air bubbles trapped in the mesenteric fat
Free pneumoperitoneum	Free extraintestinal air
Small haemoperitoneum (≤200 mL)or abundant haemoperitoneum (>200 mL)	High attenuation peritoneal fluid with a density of 35−60 HU [21]
Mesenteric/pericolic fat stranding	Streaky oedematous infiltration of the mesenteric/pericolic fat
Non-haematic free pelvic fluid	Low attenuation peritoneal fluid with a density < 25 HU
*Signs of other trauma*	
Anterior abdominal wall injury	Streaky fat infiltration of the anterior/anterolateral abdominal wall [11]
Concomitant organ injury	Injury of other solid organs, such as spleen, liver, kidneys, adrenals, and pancreas

The two radiologists jointly scored each evaluated CT examination according to two recently validated injury scores, the Faget score [11] and the BIPS [12]. To determine the BIPS, patients’ clinical records were reviewed, and each CT examination was analysed in view of any mesenteric haematoma, defined as a well-defined high-attenuating fluid collection (measuring >35HU) located within the mesenteric fat.

The Faget score is based on nine CT findings that are independently associated with sBBMI. An injury score ranging from 1 to 5 is attributed to each of the nine CT findings, as follows: bowel wall discontinuity (BWD) = 5, mesenteric pneumoperitoneum (MPP) = 5, mesenteric (pericolic) fat stranding (MFS) = 2, anterior abdominal wall injury (AAWI) = 2, active mesenteric bleeding (AMB) = 3, abundant haemoperitoneum (HP; > 200 mL) = 3, small HP (≤200 mL) = 1, and decreased bowel wall enhancement (DBWE) = 1. If these items are detected on the CT images, they need to be scored accordingly. In case of concomitant splenic injury, this numerical score needs to be reduced by 1. Whenever the Faget score accounts for ≥5 points, the high likelihood of sBBMI indicates immediate surgery [11].

The BIPS combines two physiological variables and one CT variables, with one point awarded for the presence of each (maximum score of 3). Physiological variables are the presence/absence of abdominal tenderness and level of the white blood cell (WBC) count (1 point if > 17 g/l, 0 points if lower) upon arrival in the emergency department. The CT variable describes the degree of mesenteric bowel injury on a 5-grade scale: grade 1, isolated mesenteric contusion without associated bowel wall thickening (BWT) or adjacent interloop fluid collection; grade 2, mesenteric haematoma < 5 cm without associated BWT or adjacent interloop fluid collection; grade 3, mesenteric haematoma > 5 cm without associated BWT or adjacent interloop fluid collection; grade 4, mesenteric contusion or haematoma (any size) with associated bowel wall thickening or adjacent interloop fluid collection; grade 5, AMB, BWD, or pneumoperitoneum. Grades 1–3 are scored 0 points, and grades 4–5 are score as 1 point. A BIPS of ≥2 has a sensitivity of 85.7 %, specificity of 76.2 %, positive predictive value (PPV) of 70.6 %, and a negative predictive value (NPV) of 88.9 % for the prediction of sBBMI [12].

After image analysis, the physiological items required for the BIPS and patients’ outcome were retrieved from the patients’ medical records. In case of non-documented information, a score of zero was awarded.

The two readers defined sBBMI as blunt bowel or mesenteric injury requiring timely surgical or radiological interventional treatment or that was proven by autopsy. A Faget score ≥ 5 and a BIPS ≥ 2 were considered indicative of sBBMI.

### 2.3 Statistical analysis

Statistical tests were performed using the commercially available software R [22]. Data are presented as numbers and relative percentages. Categorical variables were compared using Chi-squared and continuous variables using the student’s test or the analysis of variance (ANOVA). The Pearson correlation coefficient was used to measure the linear relationship between two continuous variables. We performed univariate and multivariate logistic regression analysis to compare the two injury scores. A p-value <0.05 was considered significant. For the problem of multiple testing, the p-values were adjusted using the Bonferroni correction.

## 3 Results

### 3.1 Patients

Of the final 752 patients, 27 % (n = 200) were women and 73 % were men (n = 552). The mean age of the whole cohort was 39.4 ± 19 years (range, 16–94 years). The sBBMI was confirmed in 21 patients (2.8 %; Table 2). In one patient, who died immediately after CT, surgery could not be performed, but forensic autopsy confirmed sBBMI. Seventeen patients underwent immediate surgery, either laparoscopy (n = 5) or laparotomy (n = 12), two of them died shortly after laparotomy. Two patients were successfully treated with angioembolisation; one of these cases was complicated by subsequent colonic ischaemia, requiring surgery. In one patient angiography found no active bleeding, and he survived with non-operative treatment.

**Table 2:** Characteristics of the 21 patients with confirmed sBBMI.

Patients	CT findings			Trauma scores		Surgical/radiological interventional findings			Delay between CT examination and surgical treatment/intervention, h
No.	Bowel and mesentery	Abdominal wall injury	Solid organ injury	McNutt score	Faget score	Bowel and mesentery	Solid organ injury	Surgical or radiological intervention	
1*	Small haemoperitoneum			1	1	None	None	Forensic autopsy	None
2	Small haemoperitoneum, diffuse SB wall thickening, mesenteric fat stranding, mesenteric vascular extravasation			2	8	Pseudoaneurysm with active mesenteric bleeding	None	Embolisation of left colic artery	3.12
3	Abundant haemoperitoneum, mesenteric fat stranding			0	8	Mesenteric laceration	None	Laparoscopy: mesenteric suture	2.4
4	Free pneumoperitoneum, small haemoperitoneum, non-focal SB thickening, mesenteric fat stranding		S	3	7	Seromuscular colon injury	Spleen	Laparotomy: colon suture, splenectomy, diaphragm suture	9.6
5	Abundant haemoperitoneum, non-focal SB wall thickening, mesenteric fat stranding, mesenteric vascular extravasation			1	10	Bleeding mesenteric vessel, SB perforation	None	Laparotomy: mesenteric suture, small bowel suture	0.72
6	Free pneumoperitoneum, mesenteric pneumoperitoneum, mesenteric vascular extravasation, small haemoperitoneum, non-focal SB wall thickening, focal colonic wall thickening, pericolic fat stranding, mesenteric fat stranding		S	3	12	Colonic and SB perforation	Spleen, kidney	Laparotomy: colectomy, small bowel suture	0.96
7*	Free pneumoperitoneum, mesenteric pneumoperitoneum, abundant haemoperitoneum, diffuse colic wall thickening, mesenteric/pericolic fat stranding, mesenteric vascular extravasation, irregular beading of mesenteric vessels			2	10	Bleeding mesenteric vessel, colon perforation	Spleen, liver	Laparotomy: colon suture, mesenteric suture, splenectomy, liver packing	0.72
8	Small haemoperitoneum, focal SB wall thickening, mesenteric fat stranding, mesenteric vascular extravasation			2	8	Bleeding mesenteric vessel	Kidney	Laparoscopy: mesenteric suture	2.16
9	Small haemoperitoneum, focal SB wall thickening, mesenteric/pericolic fat stranding			3	5	Colonic perforation *(detected on second CT examination)*	Spleen	Laparotomy: colectomy, small bowel suture	14.4
10	Small haemoperitoneum, focal absent colic wall enhancement, mesenteric/pericolic stranding, mesenteric vascular extravasation, abrupt termination of mesenteric vessels			2	7	Active mesenteric bleeding	None	Embolisation of a branch of the inferior mesenteric artery	2.88
11	Free pneumoperitoneum, mesenteric pneumoperitoneum, small haemoperitoneum, diffuse small bowel wall thickening, mesenteric/pericolic stranding, free pelvic fluid			2	10	Confirmation of absence of intestinal perforation	None	Exploratory laparoscopy: no intestinal perforation, lavage-drainage	4.32
12	Small haemoperitoneum, diffuse SB wall thickening, mesenteric vascular extravasation, mesenteric stranding			3	8	Bleeding mesenteric vessel, mesenteric haematoma	None	Laparotomy: mesenteric suture	1.2
13	Free pneumoperitoneum, focal SB wall thickening, diffuse colic wall thickening, mesenteric/pericolic stranding, free pelvic fluid	Yes	L	2	3	Colic perforation	None	Laparotomy: colectomy	2.88
14*	Abundant haemoperitoneum, mesenteric vascular extravasation	Yes	S, L, AG	2	7	Large mesenteric haematoma	None	Laparotomy: lavage	25.2
15	Free pneumoperitoneum, small haemoperitoneum, diffuse SB wall thickening, focal colic wall thickening, mesenteric/pericolic stranding, free pelvic fluid			3	5	Small bowel perforation, mesenteric haematoma	None	Laparoscopy: small bowel resection	0.48
16	Small haemoperitoneum, focal SB wall thickening mesenteric/pericolic fat stranding, mesenteric vascular extravasation	Yes	L	2	7	No active mesenteric bleeding		Angiography: no active bleeding	1.92
17	Mesenteric stranding			1	2	Mesenteric injury with secondary SB ischaemia *(detected on second CT examination)*	None	Laparotomy: small bowel resection	55.68
18	Abundant haemoperitoneum, diffuse SB wall thickening, mesenteric/pericolic fat stranding	Yes		3	6	SB perforation, bleeding mesenteric vessel	Spleen	Laparotomy: small bowel resection	73.92
19	Free pneumoperitoneum, mesenteric pneumoperitoneum, SB discontinuity, focal SB wall thickening, mesenteric/pericolic stranding, mesenteric vascular extravasation, free pelvic fluid			1	12	SB perforation	None	Laparotomy: small bowel suture	3.6
20	Free pneumoperitoneum, mesenteric pneumoperitoneum, diffuse SB wall thickening, mesenteric/pericolic stranding, free pelvic fluid			2	9	SB perforation *(detected on second CT examination),* mesenteric haematoma	None	Laparoscopy: small bowel resection	26.88
21	Small haemoperitoneum, mesenteric/pericolic stranding, mesenteric vascular extravasation	Yes	L	2	7	Bleeding mesenteric vessel, mesenteric haematoma	Liver	Laparotomy: mesenteric suture, liver packing	5.52

*Deceased.

sBBMI, significant blunt bowel mesenteric injury; M, male; F, female; SB, small bowel; S, spleen; L, liver; AG, adrenal glands.

**Faget score:** Surgical procedure needed if ≥ 5 pts. **McNutt score:** Surgical procedure needed if ≥ 2 pts.

The mean delay between CT and intervention was 11.9 h (median 3 h; range, 0.5–73.92 hours). In five patients, surgical exploration was delayed >10 h after CT. The Faget score and the BIPS were false negative in one patient, but true positive in the other four. In four of these five patients, a second CT examination was performed prior to surgery, revealing intestinal perforation twice and secondary intestinal ischaemia once. Finally, in two of these five patients, the delay in surgery could have been avoided because the initial CT showed free pneumoperitoneum (FPP) and MPP in one and AMB in the other but was not immediately reported by the on-call radiologist. The latter patient had a fatal outcome.

Of the remaining 731 patients (97.2 %) without sBBMI, 13 (1.6 %) underwent surgery for solid organ injury, confirming the absence of simultaneous sBBMI. The remaining 719 patients (95.6 %) were managed conservatively, and the absence of sBBMI was proven by uneventful clinical follow-up (>24 h).

### 3.2 CT findings

The incidence, relationship with sBBMI, and diagnostic value of the evaluated CT signs occurring in our study population are shown in Table 3. Seven evaluated CT signs were significantly associated with sBBMI (p < 0.0001): non-focal BWT, AMB, MPP, FPP, small HP, MFS, and AAWI.

**Table 3:** The incidence and diagnostic value of evaluated CT signs and the relationship with sBBMI.

CT sign	Incidence in all patients (n = 752)	Incidence in patients *without* sBBMI (n = 731)	Incidence in patients *with sBBMI* (n = 21)	p-value	Sens (%)	Spec (%)	PPV (%)	NPV (%)	Acc (%)
*Intestinal signs*									
Bowel wall discontinuity	1 (0.1 %)	0	1	0.07	4.8	100	100	97.3	97.3
Focal bowel wall thickening (≤10 cm length)	119 (15.8 %)	112	7	0.86	33.3	84.7	5.9	97.8	83.2
Non-focal bowel wall thickening (>10 cm length)	72 (9.6 %)	63	9	<0.0001	42.9	91.4	12.5	98.2	90.0
Focal decreased bowel wall enhancement	3 (0.4 %)	2	1	2.30	4.8	99.7	33.3	97.3	97.1
*Mesenteric vessel signs*									
Active mesenteric bleeding	11 (2.8 %)	0	11	<0.0001	52.3	100	100	98.7	98.7
Irregular beading of mesenteric vessels	1 (0.1 %)	0	1	0.07	4.8	100	100	97.3	97.3
Abrupt vessel termination	1 (0.1 %)	0	1	0.07	4.8	100	100	97.3	97.3
*Extraintestinal signs*									
Mesenteric pneumoperitoneum	4 (0.5 %)	0	4	<0.0001	19	100	100	97.7	97.7
Free pneumoperitoneum	9 (1.1 %)	2	7	<0.0001	33.3	99.7	77.8	98.1	97.9
Small haemoperitoneum (≤200 mL)	69 (9.2 %)	57	12	<0.0001	57.1	92.2	17.4	98.7	91.2
Abundant haemoperitoneum (>200 mL)	52 (6.9 %)	47	5	0.13	23.8	93.6	9.6	97.7	91.6
Mesenteric/pericolic fat stranding	163 (21.7 %)	144	19	<0.0001	90.5	80.3	11.7	99.7	80.6
Free pelvic fluid	64 (8.5 %)	59	5	0.50	23.8	91.9	7.8	97.7	90.0
*Signs of other trauma*									
Anterior abdominal wall injury	23 (3.1 %)	18	5	<0.0001	23.8	97.5	21.7	97.8	95.5
Solid organ injury	163 (21.7 %)	154	9	0.41	30.0	87.4	6.4	97.8	85.9

Significant p-values are in bold.

sBBMI, significant blunt bowel and mesenteric injury; Sens, sensitivity; Spec, specificity; PPV, positive predictive value; NPV, negative predictive value; Acc, accuracy.

AMB, MPP, BWD, irregular beading of mesenteric vessels (IBMV), and abrupt termination of mesenteric vessels (ATMV) were detected only in patients with sBBMI resulting in a specificity and PPV of 100 %. However, they rarely occurred (n = 1–11). Thus, the sensitivity was quite poor. In addition, BWD, IBMV, and ATMV even occurred only once, explaining the lack of statistical significance.

Although the CT signs focal BWT, MFS, and AAWI were significantly associated with sBBMI, they also occurred in patients without sBBMI. The most frequently observed CT signs in the 21 sBBMI patients were MFS (n = 19), small HP (n = 12), and AMB (n = 11), and they had the best sensitivity (90.5 %, 57.1 %, and 42.1 %, respectively) and NPV (99.7 %, 98.7 %, and 98.6 %, respectively) of all evaluated CT signs.

### 3.3 Diagnostic value of the Faget score and BIPS

The Faget score could be evaluated in all patients without any missing items. For the BIPS, all radiological findings could be collected, but information regarding WBC and abdominal tenderness was lacking in 51 (6.8 %) and 27 (3.6 %) patients, respectively. Both scores significantly correlated with sBBMI (p < 0.0001).

**Fig. 2:**
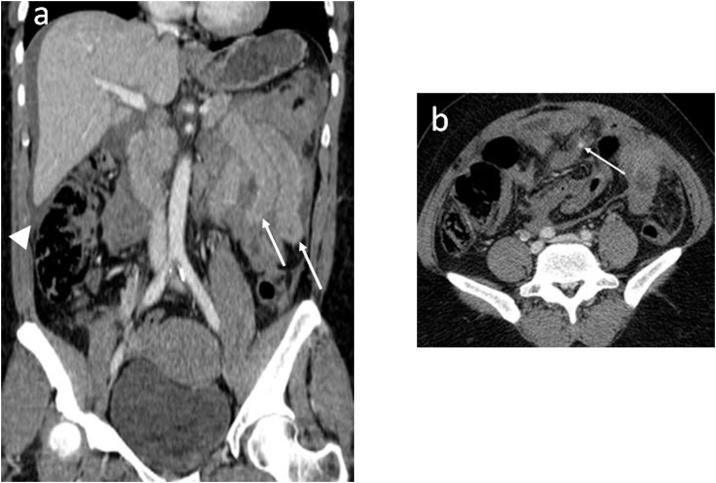
Coronal (a) and axial (b) iodinated contrast-enhanced CT images acquired in a 54-year-old woman after a traffic road accident show haemoperitoneum without solid organ laceration (a, arrowhead), non-focal jejunal bowel wall thickening (a, arrows), and mesenteric vascular extravasation (b, arrow), suggesting sBBMI. This was confirmed by immediate laparotomy (mesenteric suture, small bowel suture). McNutt and Faget scores were 1 (negative) and 10 points (positive), respectively.

The Faget score identified 18 of the 21 patients (85.7 %), and the BIPS identified 16 (76.2 %) out of the 21 patients as having sBBMI (Table 2), leading to a false negative Faget score in three patients and false negative BIPS in five patients. Both scores were false negative in the same two patients. The first patient immediately died after CT and forensic autopsy confirmed sBBMI, even though CT images only showed a small HP. In the second patient, the initial CT examination demonstrated MFS only, but a second CT examination 2 days later revealed secondary mesenteric ischaemia, leading to surgery with a delay of 55 h. The third and last patient with a false negative Faget score underwent surgery for colic perforation. However, the CT images demonstrated AAWI, liver contusion, and FPP, resulting in a Faget score of 3. The remaining three patients with sBBMI and false negative BIPS were a case with mesenteric laceration but without mesenteric haematoma >5 cm on CT **(**Fig. 2**),** and two cases with active mesenteric bleeding, but negative physiological findings (i.e., WBC < 17 g/l) - the abdominal tenderness could not be evaluated because of intubation and sedation.

Among the 732 patients without sBBMI, the Faget score was false positive in 66 patients (9%) and the BIPS in 74 patients (10.1 %).

Multivariate logistic regression analysis performed to directly compare the risk of injury estimated by the two scoring systems revealed a superiority of the Faget score to the BIPS (Table 4). The Odds ratio for the BIPS (3.16) was below the confidence interval of the Faget score (5.04−88.84). Similarly, the odd’s ratio for the Faget score was beyond the confidence interval of the BIPS.

**Table 4:** Multivariate logistic regression analysis directly comparing the risk of injury estimated by the two scoring systems.

Score	OR	95 % CI	p-value
Faget score (≥5)	18.33	5.04−88.84	<0.0001
BIPS (≥2)	3.16	1.72−6.26	<0.0001

OR, odds ratio; CI, confidence interval; BIPS, Bowel Injury Prediction Score.

## 4 Discussion

In our population of 752 polytrauma patients, 21 (2.8 %) had sBBMI confirming the low incidence of this type of injury [15,16,[1], [2], [3]] and stressing the challenge in diagnosis. In addition, initial CT signs can be subtle and non-specific. Mesenteric fat stranding may be the only sign indicative of mesenteric laceration, as shown in two of our patients with sBBMI. Timely diagnosis and early management of these cases is necessary to avoid increased morbidity and mortality [15]. Thus, the use of scoring systems is encouraged to improve the timely diagnosis of sBBMI over CT alone. The BIPS and Faget scores can augment the triaging capability of CT, as demonstrated in our study population (p < 0.001). Multivariate logistic regression analysis revealed the superiority of the Faget score to the BIPS. Although McNutt et al. concluded that patients with a BIPS ≥ 2 have a 19-times higher risk of sBBMI than patients with a BIPS < 2 [12], the BIPS applied to a retrospective series of 16 patients with sBBMI had a sensitivity of only 56.3 % [3].

The BIPS not only includes a CT grading scale, but also requires two physiological items. According to McNutt et al., a WBC count of >17 g/l and abdominal tenderness are relevant factors significantly associated with bowel injury [12]. However, as previously reported [23], no information about abdominal tenderness was available in 27 (3.6 %) of our patients. The reasons for this missing information include intubation and sedation, spinal cord injury with loss of sensation, and influence of intoxicating substances (alcohol and drugs), making a physical examination unreliable. Furthermore, the initial WBC count was not available in 51 (6.8 %) of patients, thus possibly leading to an underestimated BIPS in some cases. However, this was not relevant in our 21 sBBMI patients, as the WBC count was missing for only one patient with a false negative BIPS of 0, so that even with a WBC count >17 g/l, this BIPS would not have become indicative of sBBMI. Unfortunately, Mc Nutt et al. did not address the problem of potentially missing clinical items [12].

Two previous series could even not confirm the utility of WBC count in predicting significant blunt intestinal injury [6,24]. Similarly, several authors have questioned the utility of an initial physical examination for the diagnosis of sBBMI [10,23,25]. According to the results from the largest single-centre series to date, which included 2912 blunt abdominal trauma patients, Joseph et al. reported a sensitivity of 53 % and a specificity of 69 % for abdominal tenderness, which was inferior to CT, which had a sensitivity and specificity of 86 % and 88 %, respectively [23]. Furthermore, abdominal pain/tenderness is an important finding when present, as it may indicate intra-abdominal injury, but does not necessarily indicate surgery [23].

Unlike the BIPS, the Faget score is based on nine CT signs obtained from the initial polytrauma CT examination and has a sensitivity and specificity of 96.4 % and 91.5 %, respectively [11]. The Faget score includes not only the most specific and the most common CT signs for sBBMI, but also other indirect indicators, such as anterior abdominal wall injury, also called the “seat belt sign” [6], which we found to be significant. In the study by Faget et al., bowel wall discontinuity and mesenteric pneumoperitoneum had the strongest association with sBBMI, which compares favourably with our specificity and PPV of 100 %. Although both free and mesenteric pneumoperitoneum were significant in our study, free pneumoperitoneum was not specific for sBBMI. Free pneumoperitoneum may be caused by other trauma mechanisms, such as a pneumothorax extending into the peritoneum or a bladder rupture, and as such is excluded from the Faget score [11]. Unlike our results, the Faget score gives a higher priority to abundant haemoperitoneum (3 points) than to small haemoperitoneum (1 point). We found only the latter to be significant for sBBMI, possibly because abundant haemoperitoneum is more typical of solid organ injury.

In agreement with previous series [3,23], mesenteric fat stranding was our most frequently observed CT sign and was associated with sBBMI; however, being non-specific, its reliability in indicating a need for immediate surgery alone is limited [11,26]; thus, it must be combined with other, more specific CT signs.

Non-focal bowel wall thickening was also associated with sBBMI in our study, but focal decreased bowel wall enhancement and focal bowel wall thickening were not, stressing the difficulty in correctly analysing the intestinal wall in the context of polytrauma. For example, haemoperitoneum related to solid organ injury may for instance cause adjacent focal BWT.

CT findings previously described as being specific for sBBMI [2,10,11,16,27], such as active mesenteric bleeding, mesenteric pneumoperitoneum, bowel wall discontinuity, irregular beading of mesenteric vessels, and abrupt termination of mesenteric vessels, were also pathognomonic in our study. Thus, no false positive results occurred. Unfortunately, we observed the latter three only once, explaining the poor sensitivity and absence of statistical significance.

Due to high diagnostic value, CT is undeniably the imaging tool of choice in haemodynamically stable polytrauma patients [28], with a sensitivity of 80–95 % and specificity of 48–96 % for detecting sBBMI [16,23,27,29]. However, despite technical advancements, false negative results occur [3,6,8,10,27,30]. Although none of our 21 sBBMI patients had a strictly normal CT result, we detected non-specific CT signs only, such as mesenteric fat stranding with/without haemoperitoneum, in five (23.8 %) of them. Thus, a high index of suspicion is essential to decrease the incidence of missed blunt hollow viscus injury. Localised mesenteric fat stranding or mesenteric haematoma should prompt careful examination of the adjacent bowel loops, as extra-intestinal signs may be more striking than the underlying bowel injury. Finally, one should consider repeating the abdominal CT in selected patients, when initial images are unimpressive, but clinical signs remain worrisome [20,29], as in four of our patients with sBBMI.

The retrospective character of our study, by definition, includes documentation bias, such as missing WBC counts and information on abdominal pain. Despite the high number of included patients, only a few positive cases were found, due to the low sBBMI prevalence in general. This may somewhat limit the significance of our statistical results. In addition, the incidence of some specific CT signs was too low to become statistically significant. Furthermore, our abdominal CT acquisition was performed only in venous phase according to our routine polytrauma protocol because venous phase has been shown to be superior for active bleeding compared to the arterial phase [17,18]. Finally, non-significant BBMI may have been present in several patients who were treated conservatively, especially in patients with only non-specific mesenteric fat stranding. These patients may have had a superficial bowel tear or mesenteric haematoma without active bleeding and could be managed non-operatively.

## 5 Conclusion

The prevalence of sBBMI in polytrauma patients is low, but early diagnosis is necessary to avoid increased morbidity and mortality. Certain CT features are pathognomonic; however, they rarely occur, and early CT signs are often subtle and non-specific. Therefore, CT-based scoring systems are helpful, especially those based on radiological findings. Prospective studies are needed to better define the role of physiological parameters in sBBMI.

## CRediT authorship contribution statement

“I confirm that all the authors have made a significant contribution to this manuscript, have seen and approved the final manuscript, and have agreed to its submission to the *European Journal of Radiology Open*”

## Declaration of Competing Interest

The authors declare that they have no potential conflicts of interest regarding this study.
